# Lanthanide-Doped SPIONs Bioconjugation with Trastuzumab for Potential Multimodal Anticancer Activity and Magnetic Hyperthermia

**DOI:** 10.3390/nano10020288

**Published:** 2020-02-08

**Authors:** Weronika Gawęda, Magdalena Osial, Michał Żuk, Marek Pękała, Aleksander Bilewicz, Pawel Krysinski

**Affiliations:** 1 Institute of Nuclear Chemistry and Technology, Dorodna 16 Str., 03-195 Warsaw, Poland; W.Maliszewska@ichtj.waw.pl (W.G.); a.bilewicz@ichtj.waw.pl (A.B.); 2 Faculty of Chemistry, University of Warsaw, Pasteura 1 Str., 02-093 Warsaw, Polandpekala@chem.uw.edu.pl (M.P.)

**Keywords:** superparamagnetc iron oxide nanoparticles, SPIONs, trastuzumab, magnetic hyperthermia, antitumor therapy

## Abstract

Iron oxide-based nanoparticles have been modified in their core with holmium(III) in an amount affecting only slightly their magnetic properties. Nanoparticles were conjugated covalently with biomolecule of trastuzumab (Herceptin^®^), the monoclonal antibody that recognizes cancer cells overexpressing HER2 receptors targeting such nanoparticles to the specified tumor tissues. Systematic studies of Ho^3+^-doped bioconjugates were carried out as a preliminary step for future replacement of ‘cold’ Ho with ^166^Ho radionuclide, emitting ‘soft’ beta(-) radiation for possible targeted radionuclide therapy. Physicochemical properties of the obtained bioconjugates were subsequently tested for use in magnetic hyperthermia, considered as an effective, low-invasiveness anticancer therapy. With such a system we expect to achieve both: active targeting and multimodal action by simultaneous internal and localized irradiation and magnetic hyperthermia of specific cancers.

## 1. Introduction

Iron oxide-based magnetic nanoparticles are widely investigated because of their tunable magnetic properties and potentials in nanomedicine like targeted drug delivery [1,2], diagnostics with MRI bioimaging [3,4], and magnetic hyperthermia [5,6]. Numerous works described their therapeutic efficacy after conjugation with various drugs and biomolecules [7]. Nanosized superparamagnetic Fe_3_O_4_ nanoparticles (SPIONs) offer large surface-to-volume ratio, high surface area, easiness of modification by antitumor drugs, low toxicity, and enormous potential in targeted drug delivery and magnetic hyperthermia [8]. SPIONs have been synthesized by several methods [9] such as solvothermal and co-precipitation synthesis, combustion, thermal decomposition of precursors, reverse micelles, and sol–gel methods, however, the most common is co-precipitation, offering simple and low-cost synthesis with control of particles size, crystallinity and magnetic properties [10], features necessary for use of SPIONs for magnetic hyperthermia, i.e., the therapeutic treatment of cancer by heat generated by SPIONs under the influence of alternating magnetic field [11,12]. Although doping of SPIONs with lanthanides like Gd^3+^ or Ho^3+^, by substitution of Fe^3+^ in Fe_3_O_4_ core usually decreases magnetic properties, it can also broaden their applications in endoratiotherapy, when non-radioactive Ho cations are replaced with ^166^Ho radionuclides emitting soft, β(-) radiation [13,14]. Such lanthanide-doped SPIONs are promising candidates for multimodal effect of targeted drug delivery, combined with localized magnetic hyperthermia and endoradiotherapy [4,15]. SPIONs localized in tumor heat up in alternating magnetic field (AMF), stimulating cancer tissue destruction [16]. Due to their small size, they easily cross biological barriers [17,18]. Therapeutic hyperthermia requires optimization of nanoparticles properties, such as heating power (in terms of specific absorption rate, SAR) and concentration of nanoparticles in the target. Heating of nanoparticles up to 41–47 °C (314–320 K) for dozens of minutes by hyperthermia triggers the apoptosis [19,20], destabilizes cell and offsets homeostasis, leading to the higher susceptibility of cancer cells to chemotherapy [21]. Also, hyperthermia can potentiate the effect of radiation and has been shown to improve local treatment in patients with advanced cancers like breast [22] and head and neck [23] metastatic cancers.

The values of SAR for iron oxide-based nanoparticles depend on numerous parameters, such as size, shape, structure, composition, surface modifications, suspending medium, tissue localization, as well as AMF amplitude *H* and its frequency *f*. In the case of medical application, the product of *H* and *f* should be smaller than 5 × 10^9^ Am^−1^s^−1^ [24]. However, typical SAR values are within a few hundred watts per gram of nanoparticles [25,26].

Our previous studies presented the influence of doping with Ho^3+^ on the morphology, magnetic and structural properties of SPIONs for further modification with antitumor drugs, revealing that optimum doping of Ho(III) is between 1 and 2.5% atomic versus total iron content [27].

For the application of magnetic hyperthermia, it is very important that the nanoparticles reach tumor cells and stay in place long enough to allow a continued treatment. It can be realized through the intravenous bolus injection of magnetic nanoparticle suspension or by the attachment of the biomolecules for specific recognition of the cancer cells [28]. Similarly, as in the case of targeted therapy, the biomolecules can be monoclonal antibodies, their fragments, or smaller molecules such as amino acids and peptides. Therefore, in this paper, we report bioconjugation of Ho-doped SPIONs with HER2 monoclonal antibody trastuzumab (*Tmab*) for targeted therapy against breast cancer [29,30]. The targeting biomolecule–trastuzumab (*Tmab*) is a humanized IgG1 kappa monoclonal antibody, and is currently used to treat patients with human epidermal growth factor receptor 2 (HER2)-positive malignancies. HER2 overexpression occurs in about 20–30% of breast as well as in colon and ovarian cancers. It has been found that surface modification of nanoparticles with trastuzumab causes selectively bound to HER2 containing cells and significant internalization of the bioconjugate to the cytoplasm and deposition near the nucleus. Internalization of the bioconjugate increases the cytotoxicity of nanoparticles, especially when they contain an attached chemotherapeutic like doxorubicin [31] or radionuclide, particulary emitters of the low-range Auger electrons [32].

In vitro cytotoxicity studies of Ho-doped SPIONs were performed on the SKOV-3 cell line (ovarian cancer cells) with HER2 overexpression. This is the step of our research towards the synthesis of SPION-*Tmab* radiopharmaceutical, upon replacement of ‘cold’ Ho^3+^ with radioactive ^166^Ho^3+^ in the iron oxide magnetic core. Another issue that we addressed also in this work was whether such bioconjugation does not compromise the biological activity of trastuzumab. Thus, the presented approach shows that that the doping the magnetic core with holmium broadens the range of tentative medical applications, including endoradiotherapy when ‘cold’ holmium is replaced with ^166^Ho, emitting soft, beta(-) radiation. Moreover, bioconjugation can actively target specific cancers with multimodal action of simultaneous internal and localized irradiation and magnetic hyperthermia.

## 2. Experimental

### 2.1. Chemicals

Iron (III) chloride hexahydrate FeCl_3_·6H_2_O Aldrich ACS reagent 97%, and iron (II) chloride tetrahydrate FeCl_2_·4H_2_O puriss p.a. *≥*99% (RT), were supplied from Sigma-Aldrich (Poznan, Poland), holmium (III) chloride hexahydrate HoCl_3_·6H_2_O 99.9% trace metals was obtained from Sigma-Aldrich, 25% ammonia solution NH_4_OH was supplied from POCH. Deionized water with resistivity 18.2 MΩ cm at 25 °C was obtained using the Milli-Q ultra-pure water filtering system from Merck (Warsaw, Poland). Synthesized SPIONs were modified with 3-phosphonopropionic acid (CEPA) obtained from Sigma-Aldrich. Holmium doped SPIONs modified with CEPA were bioconjugated using the following chemicals: MES hemisodium salt (MES) from Merck with analytical grade, phosphate buffer saline (PBS) was supplied from VWR Life Sciences (VWR International, Gdansk, Poland) with ultrapure grade, EDC and sulfo-NHS from Thermo Fisher Scientific (Warsaw, Poland), analytical grade. Trastuzumab was isolated from the commercial drug Herceptin (Roche Pharmaceuticals, Basel, Switzerland).

For in vitro experiments the following materials were used: McCoy’s medium, phosphate-buffered saline (PBS), trypsin-EDTA (0.25), penicillin–streptomycin solution and fetal bovine serum from Biological Industries (Beth Haemek, Israel). The MTS solution, CellTiter 96^®^ AQueous One Solution Reagent was purchased from Promega (Mannheim, Germany). SKOV-3 cells were obtained from the American Type Tissue Culture Collection (ATCC, Rockville, MD, USA) and cultured according to the ATCC protocol.

### 2.2. Synthesis of SPIONs

**Figure 1:**
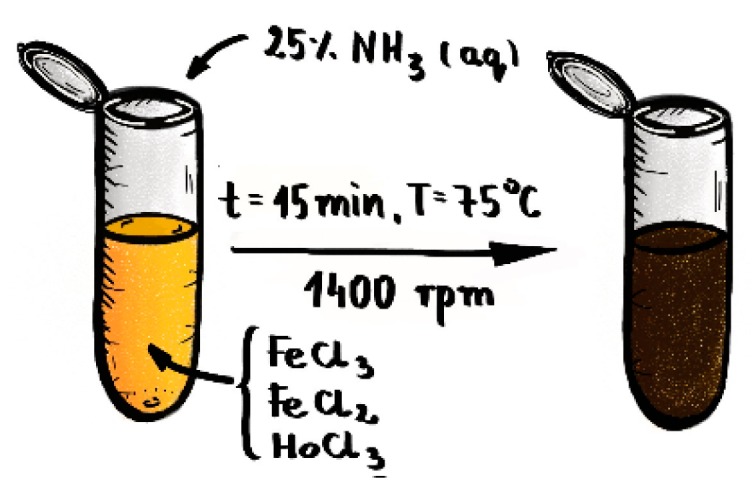
Reaction set for synthesis of Ho doped SPIONs.

The Fe_3_O_4_@1%Ho SPIONs doped with holmium were synthesized from the following solutions: 53.249 mg of ${\mathrm{FeCl}}_{3}\cdot 6H_{2}O$ in 500 μL of water, 19.881 mg of ${\mathrm{FeCl}}_{2}\cdot 4H_{2}O$ in 250 μL of water and 1.192 mg of ${\mathrm{HoCl}}_{3}\cdot 6H_{2}O$ in 200 μL of water. SPIONs were co-precipitated by mixing these solutions in 2 mL Eppendorf tube with magnetic stirrer (1400 rpm) for 2 min with the temperature set to 75 °C (348 K). Next, 120 μL of 25% NH_4_OH solution as precipitation agent was added in one bolus under mixing. Reaction was carried out for 15 min at pH 10, with constant stirring 1400 rpm. The schematic reaction set is presented at Figure 1.

The product was purified by magnetic sedimentation and rinsed with DI water until pH 7.0. Finally, SPIONs were dispersed in 1–2 mL of water.

### 2.3. Modification of SPIONs with CEPA

585.364 mg of CEPA was initially suspended in 5 mL of water in a 100 mL plastic flask. Then 90–95 mL of 0.1 M NaOH was added until pH 7. Next, 20 mg of purified Ho-SPION nanoparticles obtained in the previous step, was suspended in 1 mL of water and added drop-wise to CEPA solution while stirring. The solution was stirred for 1.5 min on magnetic stirred at RT. Next, the Ho-SPION/CEPA suspension was sonicated for 25 min at room temperature for coating reaction to finalize. Product was purified by centrifugation and rising with deionized water until pH reached 6.0.

### 2.4. Bioconjugation of SPIONs-CEPA with Trastuzumab

20 mg of dry Ho-SPION-CEPA nanoparticles was suspended in 1 mL of water in 2 mL Eppendorf tube. Twice molar excess (with respect to bound CEPA as evaluated with TGA) of EDC (2.22 mg) and sulfo-NHS (1.33 mg, bonding of –COOH groups on NPs surface) were dissolved in two separate 1.5 mL Eppendorf tubes with 500 μL of MES buffer pH 6.1. 500 μL of EDC and 500 μL of sulfo-NHS solutions were added to 2 mL Eppendorf with suspended nanoparticles. The activation reaction was performed for 4 h at room temperature in the dark. After activation, the buffer was exchanged by magnetic sedimentation/centrifugation (rinsing 3 times with 10 mM phosphate-buffered saline (PBS) buffer, pH 7.0). Finally, the activated NPs were suspended in 1.5 mL of 10 mM PBS buffer, pH 7.0 and 5 mg of trastuzumab was added. The conjugation reaction was performed overnight. The product was purified by magnetic sedimentation and rinsed 5 times with 10 mM PBS buffer pH 7.0 and finally suspended in 1.5 mL of PB buffer.

### 2.5. In Vitro Cytotoxicity Studies

In vitro cytotoxicity experiments were performed using increasing concentrations of Ho-SPION (0.78–400.00 μg/mL), Ho-SPION@CEPA (0.78–400.00 μg/mL), Ho-SPION@CEPA@Tmab bioconjugate (0.78–400.00 μg/mL) and Trastuzumab (0.78–100.00 μg/mL). SKOV-3 cells were seeded a day before experiment in 96-well microplates (TPP, Switzerland) at a density of 2.0 × 10^3^ per well. The next day, the cells were washed with PBS and treated with increasing concentrations of the compounds mentioned above, to a final volume of 100 μL per well. The cells were incubated with the compounds for 18 h at 37 °C. After this time, cells were washed twice with PBS (for removal of the unbound fraction of the compounds), and subsequently incubated at 37 °C (310 K) for 72 h. For the cytotoxicity test, the MTS assay was used. After 72 h, the MTS solution was added to each well and then incubated for another 2 h at 37 °C (310 K). Next, the absorbance of the solution in each well was measured at 490 nm using the Apollo 11LB913 microplate reader, Berthold (Bad Wildbad, Germany). Obtained results are presented as a percentage of cell viability compared to control represented by cells cultured in medium only.

### 2.6. Techniques

The size and size distribution of SPIONs were assessed with Transmission Electron Microscopy (TEM)—EF-TEM, Zeiss Libra 120 Plus, Stuttgart, Germany, operating at 120 kV. Dynamic light scattering (DLS) was used as a complementary technique to analyze the hydrodynamic size of nanoparticles. Measurements were carried out with Malvern Instruments Zetasizer Nano ZS, Malvern, UK. The magnetic properties of samples were verified with a QD vibrating sample magnetometer VSM over the magnetic field range from *−*2.0 T to +2.0 T at a temperature ranging from 100 K to 300 K with accuracy of ca. 0.01 K. Magnetization and coercive fields were measured with accuracy better than 1%. The presence of organic compounds on the SPIONs surface was investigated by FTIR spectroscopy with Nicolet 8700 Spectrometer Fisher Scientific. Thermogravimetric analysis (TGA) was performed with TGA Q50 (TA Instruments), New Castle, PA, USA, under a nitrogen atmosphere.

Magnetic hyperthermia was measured with nanoScale Biomagnetics D5 Series equipment with CAL1 CoilSet. The SAR values were estimated using MaNIaC Controller software (nB nanoScale Biomagnetics, Zaragoza, Spain).

## 3. Results and Discussion

### 3.1. Morphology

**Figure 2:**
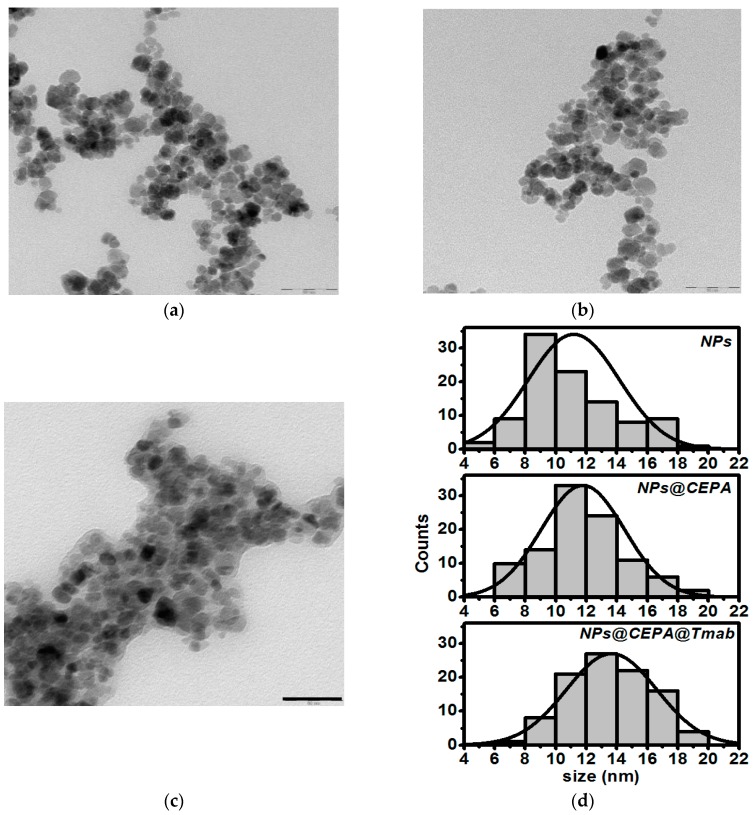
TEM image and NPs size distribution of (**a**) as-synthesizes SPIONs, (**b**) SPIONs functionalized with 3-phosophonopropionic acid, (**c**) SPIONs functionalized with trastuzumab, and (**d**) histograms based on TEM images (Scale bar: 50 nm).

TEM studies reveal an average diameter of SPIONs about 10–15 nm, see Figure 2. In the case of CEPA-modified SPIONs, the organic layer is not well visible, however, each step of the conjugation procedure seems to increase the symmetry of histogram with increasing maximum size of nanoparticles. This is not a surprising result, yet it provides evidence of successful surface modification with trastuzumab molecules. In all cases, the nanoparticles were generally spherical with apparent aggregation in the vacuum environment of TEM. These results correspond well to our previous results of Ho-doped SPIONs under similar conditions [27].

The distribution of hydrodynamic size for SPION suspension at each step of surface modification was monitored with the dynamic light scattering (DLS) analysis. As presented in Table 1, the DLS data show a larger diameter compared to that obtained by TEM analysis, and this can be attributed to the presence of the solvation layer of water. Hydrodynamic size of unmodified SPIONs is 57 ± 1.6 nm, while this value increases after CEPA functionalization and bioconjugation with trastuzumab. Additionally, the suspension stability was determined by zeta potential measurements. The negative value of this potential, increasing after each modification step shows increased stability of SPION suspension due to the repulsive Coulomb forces.

### 3.2. FTIR Analysis

**Figure 3:**
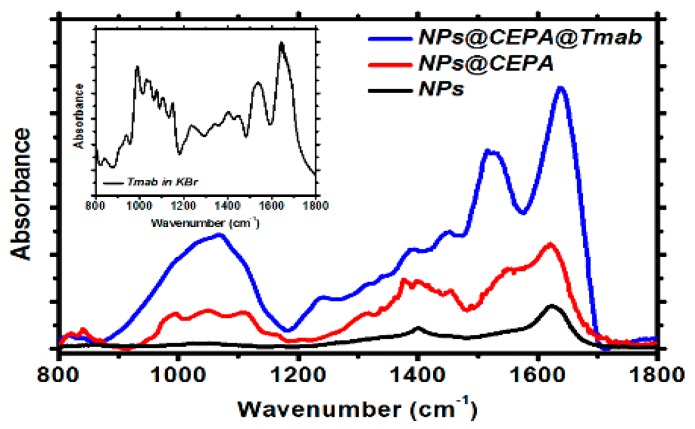
FTIR spectra for unmodified SPIONs (black curve), SPIONs modified with CEPA (red curve), and bioconjugated SPIONs (blue curve).

To confirm the presence of the organic compounds on SPION surface, the FTIR analysis was performed. Figure 3 shows the FTIR spectra (diagnostic regions) of pristine, as-synthesized SPIONs (a), modified with CEPA (b), and conjugated with Tmab through CEPA linker (c). Inset in Figure 3 presents the spectrum of Tmab in KBr alone.

Figure 3, black curve, presents a typical spectrum of magnetite with two merging bands at ca. 575 cm^−1^ and 630 cm^−1^, characteristic for the Fe-O lattice vibration [33]. The absorption band at ca. 1410 cm^−1^ and 1620 cm^−1^ can be related to the vibrations of -OH from water molecules still adsorbed on the surface, as well as surface-bound –OH groups present on the nanoferrite surface [34]. Fe_3_O_4_–CEPA (Figure 3, red curve) reveals characteristic absorption at 1630 cm^−1^, corresponding to C=O stretching vibrations in protonated–COOH group, which subsequently takes part in trastuzumab conjugation through the carbodiimide (EDC/NHS) procedure. Upon the conjugation of Tmab to the CEPA-modified nanoparticles (Figure 3, blue curve), two additional bands appeared, being a fingerprint of successful conjugation of this protein to the magnetic nanoparticles: these are the amide I stretching vibrations at 1640 cm^−1^ and amide II in-plane N-H bending together with C-N and C-C stretching, appearing at ca. 1530 cm^−1^.

### 3.3. Thermogravimetric Analysis (TGA)

**Figure 4:**
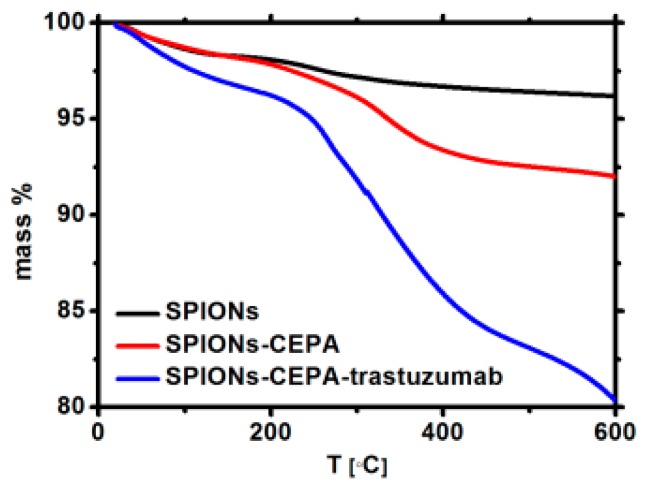
Thermograms of the SPIONs (black), SPIONs modified with CEPA (red), and bioconjugated (blue).

The TGA analyses of Fe_3_O_4_@1%Ho, Fe_3_O_4_@1%Ho@CEPA, and Fe_3_O_4_@1%Ho@CEPA@Tmab were carried out in the temperature range from ambient to 600 °C, with a heating rate of 10 °C/min, under nitrogen (Figure 4). The gradual mass loss during heating can be assigned to the decomposition of organic compounds modifying the surface of nanoparticles. The mass loss of ca. 5% for SPIONs modified with CEPA and 12% for SPIONs bioconjugated with Tmab, shows the amount of organic compounds bound to the surface. Taking into account the amount of *Fe_3_O_4_@1%Ho@CEPA* (20 mg) and Tmab (5 mg) used in the conjugation reaction, we evaluate the ‘efficiency’ of this reaction to be ca. 54.6% by mass. We are currently working on the increase of this efficiency to minimize the loss of unbound antibody molecules.

### 3.4. Magnetic Analysis

**Figure 5:**
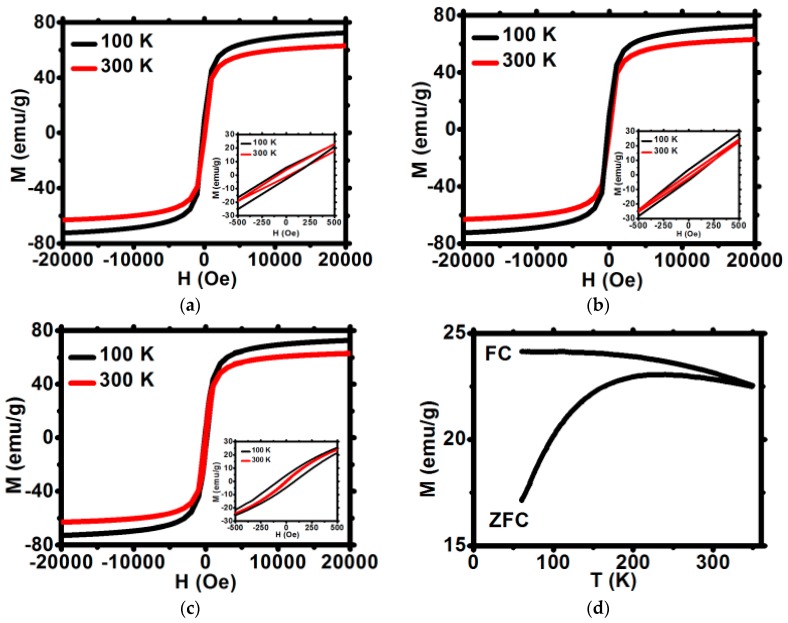
Magnetization isotherms for (**a**) Fe_3_O_4_@1%Ho, (**b**) Fe_3_O_4_@1%Ho@CEPA, (**c**) Fe_3_O_4_@1%Ho@CEPA@Tmab. All curves are normalized for the mass of NPs core. (**d**) The temperature dependence of ZFC and FC magnetization at 100 Oe for *Fe_3_O_4_@1%Ho* (**d**).

The magnetization saturation for Fe_3_O_4_@1%Ho, Fe_3_O_4_@1%Ho@CEPA, and Fe_3_O_4_@1%Ho@CEPA@Tmab were measured in a broad range of applied magnetic field. Magnetization increases abruptly up to about 1000 Oe and reaches saturation up to ca. 72 emu/g, which is very close to the values obtained for the undoped, bulk magnetite, and corresponds to our previous results [32]. As can be seen in Figure 5a–c the magnetization does not decrease after modification with organic compounds—a very advantageous feature form the point of view of SPIONs’ usage as a targeted drug delivery platform.

#### 3.4.1. Coercivity

The shape of narrow hysteresis loops plotted in Figure 5 indicates the superparamagnetic properties of SPIONs. The low field part of hysteresis loops shown in insets enables to determine the coercivity field H_C_ at 100 and 300 K. Values of coercive field H_C_ at 300 K are very close to zero and do not exceed 10 Oe at 300 K (Table 2) whereas H_C_ increases up to 50 to 80 Oe at temperature of 100 K.

These negligibly small values of H_C_ at room temperature are characteristic for superparamagnetic materials and are comparable to room temperature Hc values range between 0.55 and 9 Oe reported for similar superparamagnetic materials [35]. Thus, the first criterion for superparamagnetism is obeyed. Such small Hc values at 300 K are due to some residual inhomogeneities and local magnetic anisotropy, which are inherent for real magnetic materials. Moreover, the M(H) dependence of the studied materials was checked to follow strictly the Brillouin function. This observation confirms that the second superparamagnetism criterion is also obeyed. Furthermore, the ratio of the remnant magnetization, M_R_, to the saturation magnetization, M_S_, is as low as 0.5%, whereas for the ferromagnetic materials the M_R_/M_S_ ratio may exceed 50%. All the above features of SPIONs studied, confirm the superparamagnetism of bioconjugates [18].

#### 3.4.2. Low Field Magnetization

The low field magnetization was measured in the zero-field cooled (ZFC) and field cooled (FC) modes applying a field of 100 and 300 Oe (Figure 5d). The FC magnetization slowly decreases with temperature rise, both for 100 and 300 Oe fields. The values of FC magnetization are slightly reduced for the surface-modified samples as compared to the unmodified nanoparticles. The ZFC decrease observed at low temperature, reveals some antiferromagnetic contribution in the three samples studied. The FC and ZFC curves merge at the irreversibility temperature T_IR_ located well above the room temperature. A very shallow maximum of the ZFC magnetization curve reveals a broad distribution of blocking temperatures.

#### 3.4.3. High Field Magnetization

The magnetization versus magnetic field characteristics of the three samples studied exhibit an abrupt increase at low fields up to approximately 2 T, which is followed by the slow approach to saturation (Figure 5a–c). The high field M vs. H characteristics may be described by the formula

M = Ms (1 − b/H^2^) + cH

where Ms is the saturation magnetization. The b and c parameters correspond to the magnetic anisotropy effect and the so-called forced magnetization, respectively [36,37]. A careful fitting of experimental data to the above formula allows to determine values of Ms, as well as both parameters c and b. They are listed in Table 3 for temperatures 100 K and 300 K. Figure 5a–c show the experimental data (symbols), as well as the fitted curves (lines) with parameters listed below. Both the measured and fitted curves coincide for a broad range of fields higher than 5000 Oe. The saturation magnetization is lower than that of pure magnetite (72 emu/g) and decreases with temperature rise. Moreover, modification of the SPION surface with CEPA and Tmab diminishes further the Ms values of nanoparticles. However, assuming that parameter b is related to the nanoparticle anisotropy by

b = (8/105) (K/µ_0_ Ms)^2^

the anisotropy energy K and anisotropy field H_k_ were determined and are shown in Table 3. As can be seen, the anisotropy energy K increases by a few percent when temperature decreases from 300 down to 100 K, whereas the anisotropy field H_k_ exhibits an opposite tendency. The K and H_k_ values are comparable to those reported for similar magnetite-based compounds [36,37].

### 3.5. Magnetic Hyperthermia

To evaluate the effect of heating efficiency, the magnetic hyperthermia measurements were performed using the suspension of SPIONs without and with bioconjugation. The samples were inserted into the copper coil (thermostated). The measurements were performed with alternating magnetic field in the frequency range of 345.5–790 kHz and with the amplitude up to 350 G. Nanoparticles were dispersed into the 0.5 mL of water with a density of 10 mg/mL. The small volume was chosen to reduce the heating inhomogeneity. The time required to reach 45 °C (318 K)was monitored in order to evaluate the heating rate and SAR values of the suspension. The conversion of magnetic energy into heat in an alternating magnetic field generally proceeds through the Néel and Brownian effects [26,37,38]. In magnetic hyperthermia, the tumor temperature has to be increased locally up to the value causing changes in the physiology of the cancer cells followed by cell death.

**Figure 6:**
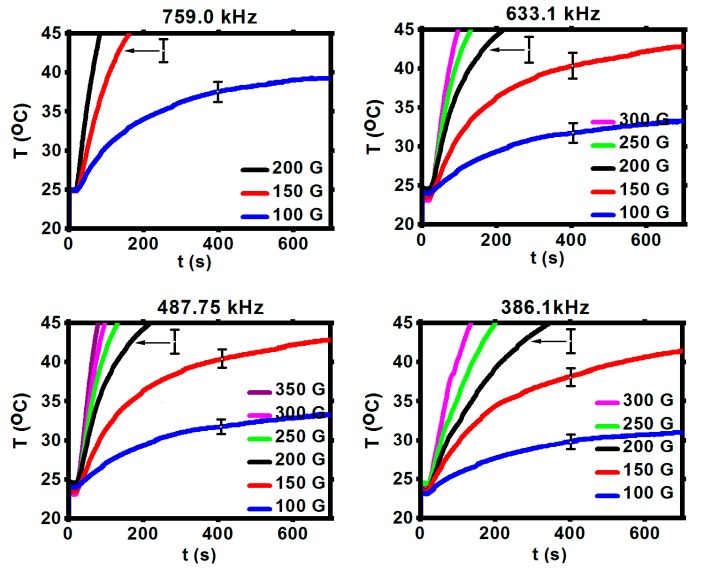
Heating of SPIONs@CEPA@Tmab in various range of frequency and magnetic amplitude. Error bars show the highest standard deviation for each magnetic flux density [G].

Figure 6 shows the changes in temperature depending on the magnetic field with various frequencies and amplitudes. Due to the equipment parameters, the amplitude values of ca. 350 G could be applied only for frequencies below 633.1 kHz. As can be seen in Figure 6, the application of high frequency 633.1–759 kHz and high amplitude caused a spontaneous rise of the suspension’s temperature. The plateau temperature is reached at lower amplitudes, so to reach 45 °C (318 K) the frequencies from 345.5 kHz up to 487.75 kHz and amplitudes 150–200 G are optimal, being within the biophysical limitations for such therapy (the so-called Brezovich limit) [16,24,38].

The values of SAR for bioconjugates are lower than for uncovered SPIONs, however, bioconjugated SPIONs are stable, whereas pure SPIONs precipitate quickly, excluding their applications for biological systems. SPIONs bioconjugated with CEPA and trastuzumab, presented within our studies, show high colloidal stability with high values of SAR making them good candidates for future use in targeted drug delivery, hyperthermia, and endoradiotherapy.

**Figure 7:**
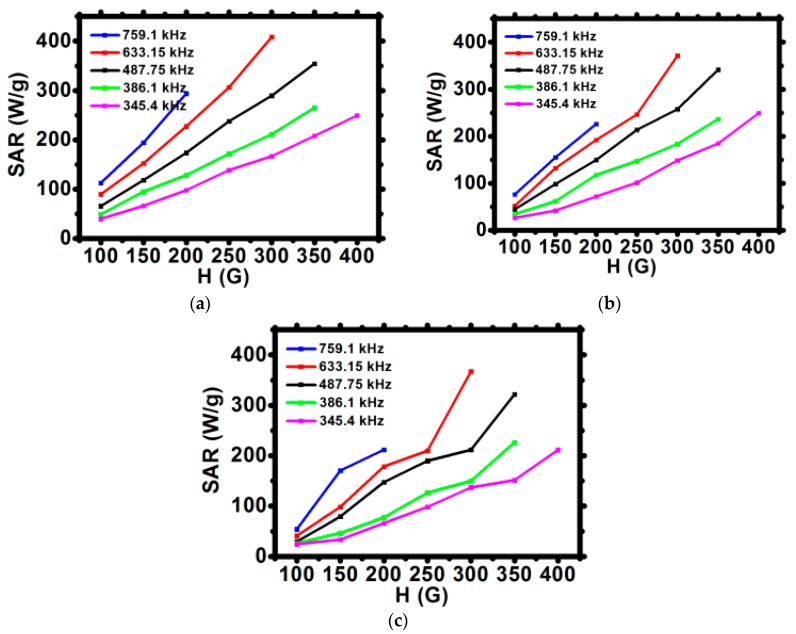
Dependence of the SAR for various frequencies of magnetic field in function of the amplitude of magnetic field H for (**a**) SPION, (**b**) SPION@CEPA, (**c**) SPION@CEPA@Tmab.

The specific absorption rate was estimated for the initial linear range of T as a function of time curves (Figure 6). The values of SAR for uncovered and surface-modified SPIONs are presented in Figure 7a–c. In an alternating magnetic field, the heat generation generally occurs due to the relaxation and hysteresis loss. Linear growth of the SAR values for uncovered SPIONs suggests the Brownian and Néel relaxation [39].

The modification of the SPIONs surface decreases the values of SAR by ca. 15%. This is evidenced by a decreasing slope of SAR vs. H graphs from Figure 7a to Figure 7c. This observation suggests that a decrease of heat dissipation for surface-modified SPIONs can be attributed to the reduction of Brownian relaxation (e.g., nanoparticle rotation) [16]. TEM images and DLS studies confirm that relatively large hydrodynamic size of bioconjugated SPIONs may influence the Brownian relaxation time, thus decreasing its contribution to the values of SAR. Nevertheless, despite slightly reduced SAR values, during magnetic hyperthermia measurements, bioconjugates showed good colloidal stability—an important issue in medical applications.

### 3.6. In Vitro Cytotoxicity Results

**Figure 8:**
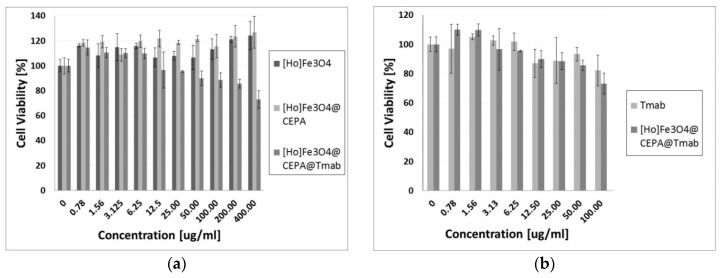
Cytotoxicity results on SKOV-3 cell line after 72 h of incubation for (**a**) Ho-SPION, Ho-SPION@CEPA, Ho-SPION@CEPA@Tmab; and (**b**) Tmab in comparison to Ho-SPION@CEPA@Tmab.

The results of cytotoxicity studies performed by the MTS assay method are presented in Figure 8. They are presented as a percentage of viability of cells treated with our bioconjugates compared to control, represented by non-treated cells cultured in the same medium only. The viability of the control group is set as 100%. It is quite often in the MTS assay that if tested substances show no toxicity at certain concentrations, the cell viability may exceed 100% [40]. This may happen due to slightly increased cell proliferation or natural variations of cellular metabolism. Also small pipetting errors may be the reason behind the higher cell viability (~120%).

The obtained results of cytotoxicity studies (Figure 8a) exhibit that Ho-SPION and Ho-SPION@CEPA have a non-toxic effect on the viability of the SKOV-3 cells. At the same time, however, Ho-SPION@CEPA@Trastuzumab bioconjugates reduced the metabolic activity of SKOV-3 cells, in a dose-dependent manner, to the 73.12 ± 7.12% for the highest concentration of the compound. This is related to the cytotoxicity of the attached trastuzumab, which, in the form of Herceptin^®^, is currently the first choice drug for HER2+ breast and ovarian cancers. Moreover, the obtained Ho-SPION@CEPA@Trastuzumab bioconjugate has even slightly higher toxicity than trastuzumab alone. This is shown in Figure 8b (the results are recalculated for the concentration of trastuzumab). Our results also indicate that the conjugation of trastuzumab to SPION nanoparticles does not compromise the activity of the monoclonal antibody. These results are in agreement with previously published paper [27], where PEG was used as a linker for attaching trastuzumab.

## 4. Conclusions

We have successfully synthesized SPIONs doped with 1 at % holmium as heat mediators for magnetic hyperthermia. We also conjugated them with monoclonal antibody, trastuzumab (*Tmab*), for targeted therapy against breast cancer with HER2 receptors without compromising the biological activity of vector molecules. Cytotoxicity studies demonstrated that Ho-SPION@CEPA@Trastuzumab bioconjugates reduced the metabolic activity of SKOV-3 cells in a dose-dependent manner. Moreover, they exhibited a slightly higher toxicity than trastuzumab alone. The bioconjugates thus formed are stable during the application of alternating magnetic field, which is a very important feature for their possible medical applications. The obtained values of specific absorption rate, SAR, reveal their potential and effectiveness in magnetic hyperthermia. Their properties prove that they should preferentially heat the cancerous tissue, whilst sparing the surrounding normal cells that do not overexpress HER2 receptors. Additionally, doping the magnetic core with holmium broadens the range of tentative medical applications, including endoradiotherapy when ‘cold’ holmium is replaced with ^166^Ho, emitting soft, beta(-) radiation. Moreover, holmium doped SPIONs conjugated with various therapeutic agents can be exploited for several applications, such as MRI imaging or fluorescence labeling. Presented results are the step of our research towards the synthesis of SPION-*Tmab* radiopharmaceutical, upon replacement of ‘cold’ Ho^3+^ with radioactive ^166^Ho^3+^ in the iron oxide magnetic core. With such a system we expect to achieve both: active targeting and multimodal action by simultaneous internal and localized irradiation and magnetic hyperthermia of specific cancers.

## Figures and Tables

**Table 1:** Values of average hydrodynamic diameter and zeta potential

Sample	Hydrodynamic Diameter (nm)	Zeta Potential (mV)
NPs	57	−1.20
NPs@CEPA	98	−30.5
NPs@CEPA@Tmab	162	−45.4

**Table 2:** Values of coercivity for NPs before and after modifications with organic compounds

Sample	H_c_ (Oe) at 300 K	H_c_ (Oe) at 100 K
Fe_3_O_4_@1%Ho	7.0	70
Fe_3_O_4_@1%Ho@CEPA	2.0	50
Fe_3_O_4_@1%Ho@CEPA@Tmab	10	80

**Table 3:** Values of anisotropy energy K in function of temperature for samples without and with organic shell.

T (K)	c (emu/g^.^Oe)	b (Oe^2^)	K (erg/g)	H_k_ (Oe)
**Fe_3_O_4_@1%Ho**				
300	1.49 × 10^−4^	3.14 × 10^6^	4.2 × 10^5^	6.42 × 10^4^
100	2.03 × 10^−4^	2.88 × 10^6^	4.43 × 10^5^	6.15 × 10^4^
**Fe_3_O_4_@1%Ho@CEPA**				
300	1.34 × 10^−4^	3.04 × 10^6^	3.81 × 10^5^	6.32 × 10^4^
100	1.75 × 10^−4^	2.96 × 10^6^	4.13 × 10^5^	6.24 × 10^4^
**Fe_3_O_4_@1%Ho@CEPA@Tmab**				
300	1.26 × 10^−4^	2.84 × 10^6^	3.31 × 10^5^	6.11 × 10^4^
100	1.57 × 10^−4^	3.01 × 10^6^	3.74 × 10^5^	5.66 × 10^4^
